# Supplementary material for: Impact of Bis-O-dihydroferuloyl-1,4-butanediol Content on the Chemical, Enzymatic and Fungal Degradation Processes of Poly(3-hydroxybutyrate)
Source: Polymers (Basel). 2022 Apr 11;14(8):1564. doi: 10.3390/polym14081564 (PMC9031392; doi:10.3390/polym14081564)
Supplement: Supplementary file 1 [file polymers-14-01564-s001.zip › polymers-1661901-supplementary.pdf]

polymers

MDPI

Supplementary Materials

Impact of Bis-O-Dihydroferuloyl-1,4-Butanediol Content on the Chemical, Enzymatic and Fungal Degradation Processes of Poly(3-Hydroxybutyrate)

Quentin Carboué *, Sami Fadlallah *, Yasmine Werghi, Lionel Longé, Antoine Gallos, Florent Allai and Michel Lopez

URD Agro-Biotechnologies Industrielles (ABI), CEBB, AgroParisTech, 3 Rue des Rouges-Terres, 51110 Pomacle, France; yasmine.werghi1@gmail.com (Y.W.); lionel.longe@agroparistech.fr (L.L.); antoine.gallos@agroparistech.fr (A.G.); florent.allais@agroparistech.fr (F.A.); michel.lopez@agroparistech.fr (M.L.)

* Correspondence: quentin.carboue@agroparistech.fr (Q.C.); sami.fadlallah@agroparistech.fr (S.F.).

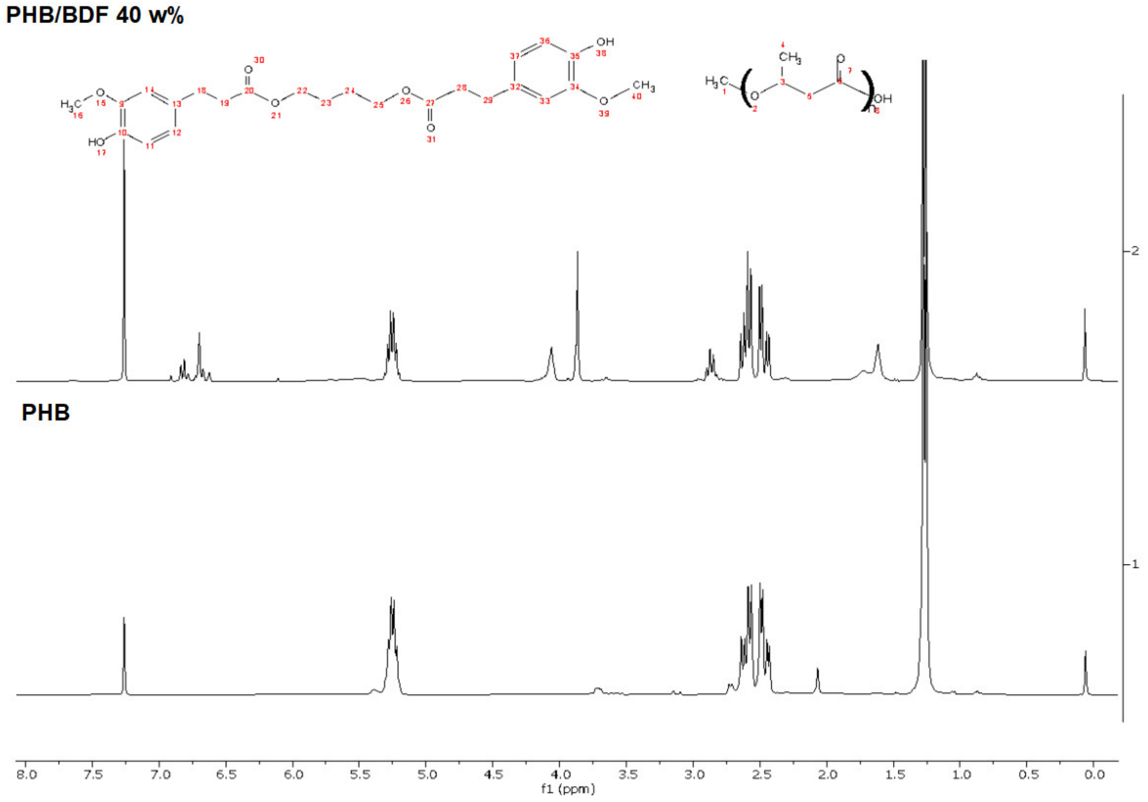

Figure S1. ^1H NMR ($CDCl_3$) spectra of PHB/BDF 40 w% and PHB.

PHB : ^1H NMR (300 MHz, $CDCl_3$, δ ppm): 5.25 (dd, *J* = 12.7, 6.3 Hz, 1H), 2.78 – 2.37 (m, 2H), 1.27 (d, *J* = 6.2 Hz, 3H).

BDF : ^1H RMN (400 MHz, $CDCl_3$, δ ppm): 6.82 (2H, d, *J* = 8, H_3), 6.68 (4H, m, $H_{4,6}$), 5.60 (2H, s large, H_1, Ar OH), 4.06 (4H, m, H_{12}), 3.85 (6H, s, H_8), 2.87 (4H, t, *J* = 8, H_9), 2.59 (4H, t, *J* = 8, H_{10}), 1.62 (4H, m, H_{13}).

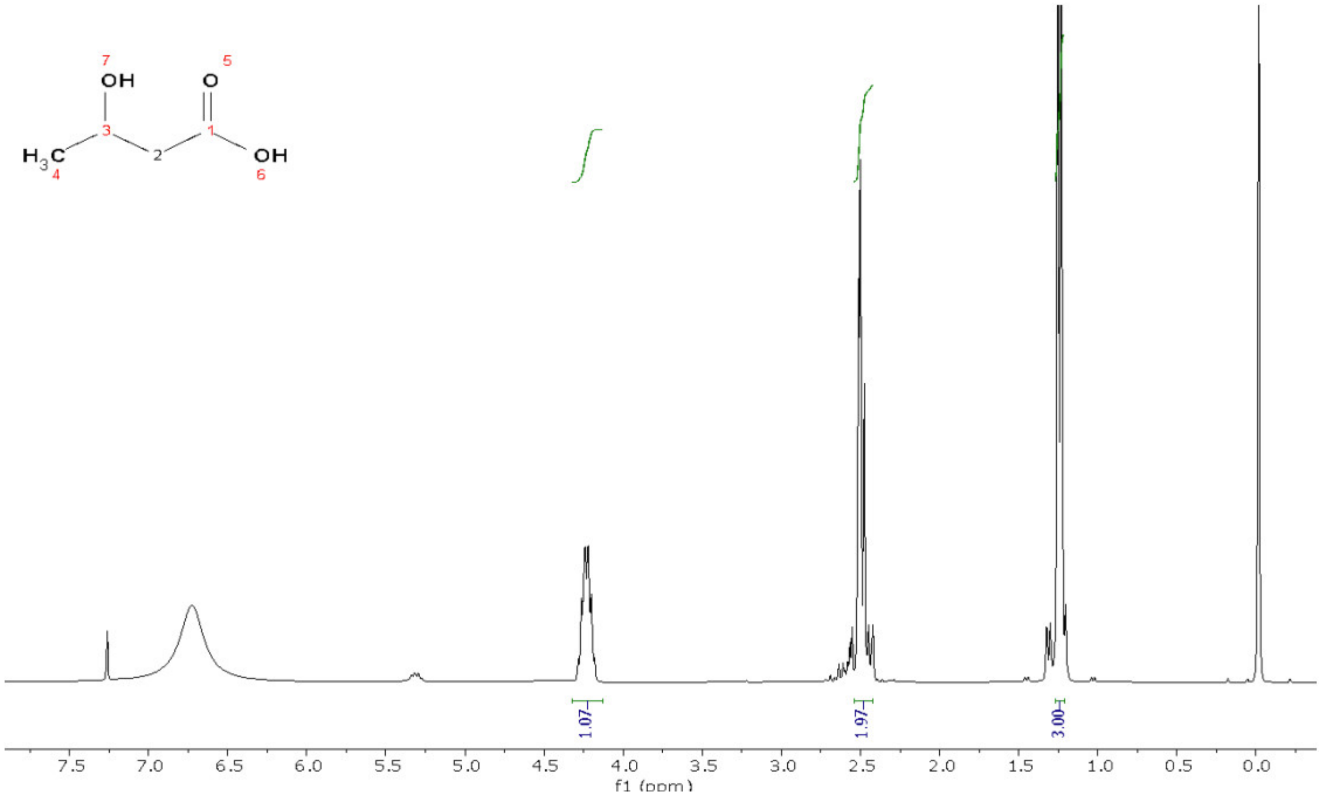

Figure S2. ^{1}H NMR ($CDCl_3$) spectrum of β-hydroxybutyric acid.

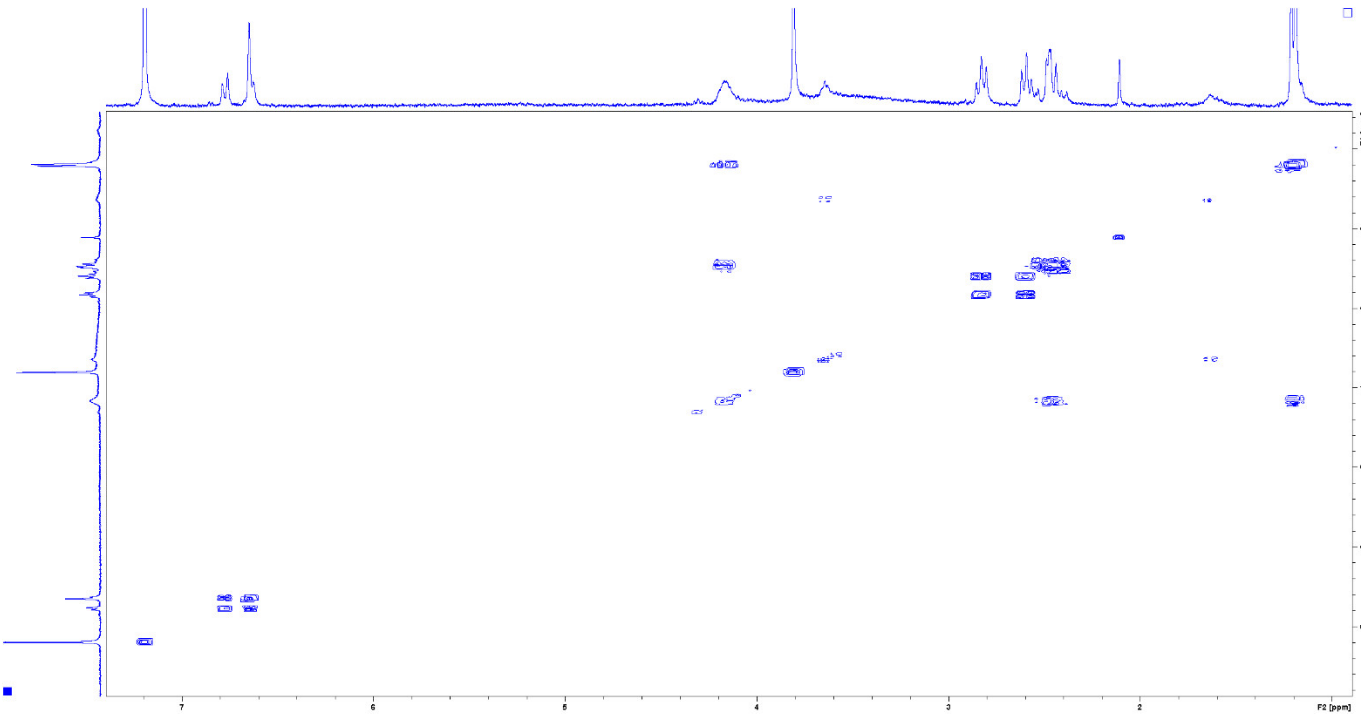

Figure S3. ^{1}H-^{1}H COSY ($CDCl_3$) of PHB/BDF (40 w%) after 32 h of degradation with NaOH solution (0.25 M).

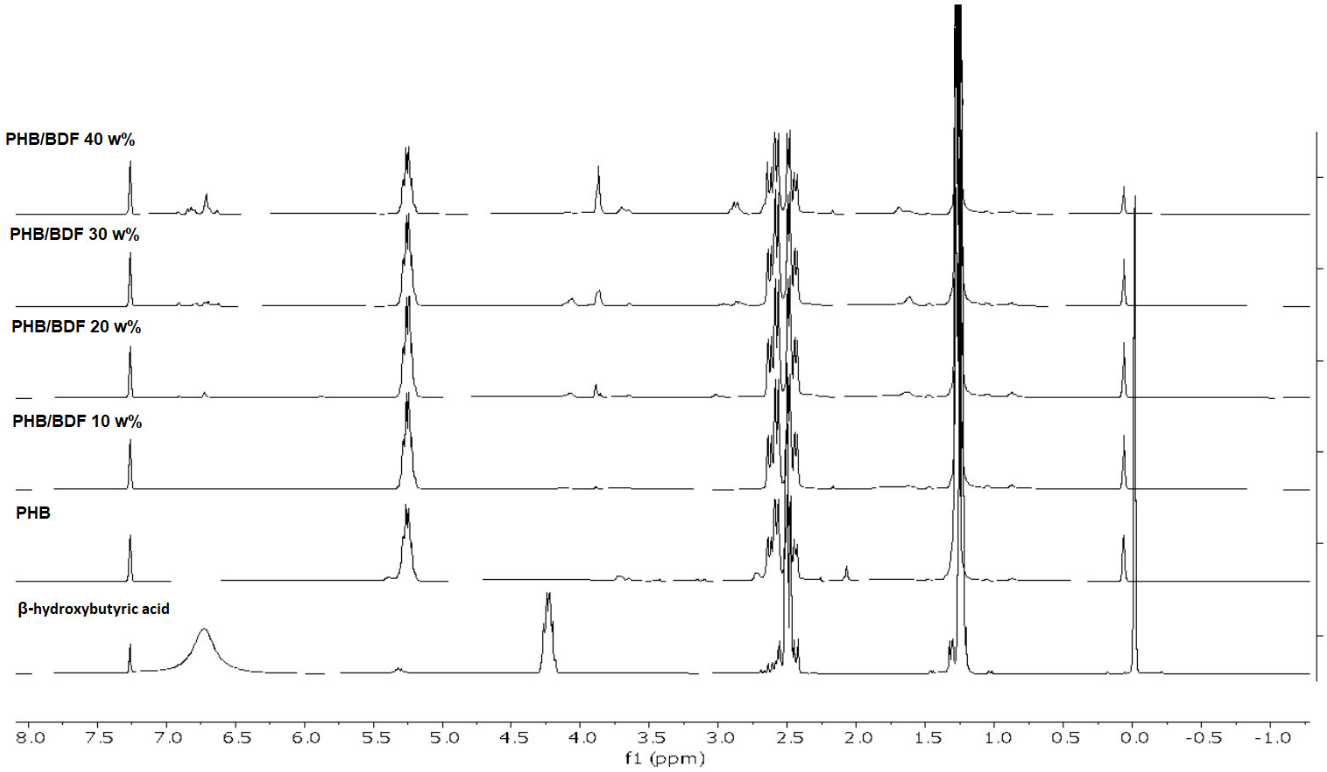

Figure S4. ^{1}H NMR ($CDCl_3$) spectra of powders of PHB blends after 7 days of reaction with 5.8 mg.mL^{-1} of Lipopan© 50 BG at pH = 7.4, 37 °C and stirred at 50 rpm.

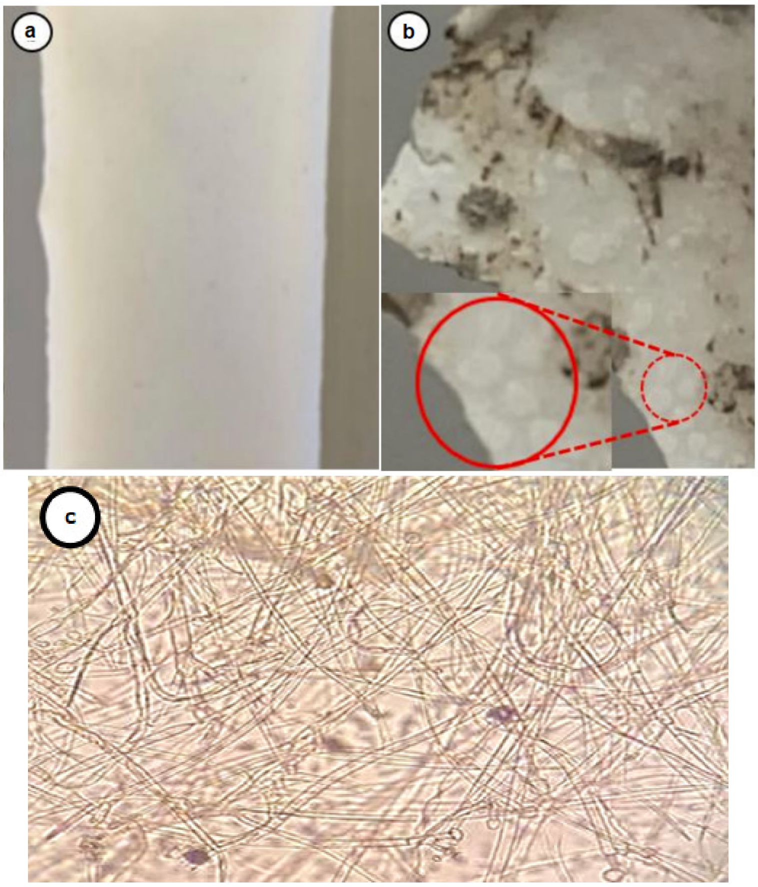

Figure S5. Pictures of the PHB film a./ at t0 and b./ after 4 weeks in the soil samples c./ microscopic observation of *A. elegans*, the aseptate hyphae and sporangia are visible.
